# Health system governance and pharmaceutical policy in Australia and Qatar: a comparative overview of access, reform, and equity

**DOI:** 10.1080/20523211.2026.2734138

**Published:** 2026-09-30

**Authors:** Dina Abushanab, Daoud Al-Badriyeh

**Affiliations:** a Office of Vice President for Health & Medical Sciences, QU Health, Qatar University, Doha, Qatar; b College of Pharmacy, QU Health, Qatar University, Doha, Qatar

**Keywords:** Healthcare system, Australia, Qatar, value-based healthcare, public, policymaking

## Abstract

This commentary contrasts the healthcare systems in Australia and Qatar, emphasising how each nation addresses shifting health policy priorities amid demographic pressures, financing reforms, and pharmaceutical challenges. Australia, with its established Medicare system and institutionalised Health Technology Assessment (HTA), continues to face issues related to equity, rural access, and the demands associated with an aging population. In contrast, Qatar is actively reforming its healthcare infrastructure to support a young and diverse expatriate-majority population, with increasing investments in insurance coverage, HTA integration, and private sector participation. Despite their differing levels of system maturity and population needs, both countries are tackling common issues: the rising burden of non-communicable diseases, cost pressures from innovative treatments, and the necessity of aligning expenditures with measurable health outcomes. The commentary highlights value-based healthcare as a shared goal while critically assessing the impact of pharmaceuticals, data infrastructure, and stakeholder engagement in promoting sustainable and equitable access. By integrating insights and policy reflections within a comparative framework, this commentary identifies opportunities for cross-national learning and outlines potential directions for future research and practice.

## Introduction

Health systems are complex entities comprising multiple interacting components (McCoy & Allotey, 2021). According to the World Health Organisation (WHO), a health system encompasses all organisations, people, and actions aimed at promoting, restoring, or maintaining health. These systems operate at the country-level, integrating both rural and urban areas, public and private sectors, and formal and informal healthcare provisions (World Health Organization and World Bank, 2002).

Health systems go beyond providing medical services and shape social well-being, economic progress, and population health outcomes (Gilson, 2003; World Health Organization and World Bank, 2002) Pharmaceuticals also play a vital role in health systems, influencing not only accessibility and treatment outcomes for populations but also shaping public health policies and economic sustainability.

This commentary compares Australia and Qatar, two high-income countries with universal health coverage but shaped by different governance, funding models, and pharmaceutical policy contexts. Australia operates a well-established health system, supported by mature health technology assessment (HTA) procedures and pharmaceutical reimbursement pathways (Australian Competition & Consumer Commission, 2023). Qatar, in comparison, is currently implementing rapid reforms to its health financing, insurance systems, and moves toward value-based care (AlRuthia et al., 2025). These distinct policy paths make it possible to assess how different approaches affect pharmaceutical availability, health equity, and overall system sustainability. Although the two countries differ, both generally deliver strong health outcomes, including life expectancy above 80 years, while still confronting ongoing issues such as demographic shifts, rising chronic disease burdens, and pressures on healthcare sustainability.

Health behaviours of the Australian and Qatari populations also differ. In Australia, 85% of individuals aged 15 and older consider their health to be ‘good’ or ‘very good’ (Australian Institute of Health and Welfare, 2018). In Qatar, 54.6% describe their health as ‘good’ and 24.7% as ‘excellent,’ (Ahmed et al., 2023), indicating a fairly high level of perceived well-being, albeit slightly lower than in Australia. Obesity is a shared challenge in both countries, but it is more pronounced in Qatar. As of 2017, 72% of Qatari adults were classified as overweight and 35% obese (‘Nutrition Country Profile. Qatar,’), compared to 34% overweight and 26% obese in Australia (Keramat et al., 2021). These trends reflect lifestyle changes due to urbanisation and sedentary behaviours in both nations, but Qatar’s higher obesity rates are particularly concerning. In terms of substance use, Australia has higher alcohol consumption, influenced by more permissive social norms (Australian Institute of Health and Welfare, 2018). Conversely, Qatar's low alcohol intake is largely due to Islamic legal restrictions, which may help mitigate alcohol-related harms. Smoking is a public health concern in both nations, though prevalence figures vary by population group and how they are measured. In Qatar, adult tobacco use has been reported at about 25%, whereas Australia’s daily smoking prevalence is much lower, highlighting the effects of long-established tobacco-control policies (AlMulla et al., 2021; Aw et al., 2024).

This commentary utilises a comparative framework based on the WHO’s health system building blocks, which encompass service delivery, health workforce, health information systems, access to essential medicines, financing, and leadership/governance (WHO, 2010). These domains were chosen for their significance in both national contexts and their compatibility with global policy evaluation tools. By organising the comparison around these thematic areas, especially pharmaceutical policy, health financing, access, and equity, this analysis facilitates a more systematic understanding of how various governance models influence health outcomes and reform pathways.

This commentary uses a comparative policy lens to explore how Australia and Qatar structure and manage pharmaceutical access through health financing, governance, and HTA. Guided by the World Health Organization (WHO) health system building blocks as an analytical framework, the paper seeks to produce policy-relevant insights into equity, access to medicines, and health system reform across two distinct high-income contexts.

## High-quality healthcare systems

High-quality healthcare systems are built on four core values: serving the people, promoting equity, demonstrating resilience, and operating efficiently (Kruk et al., 2018). Serving the people involves not only providing access to healthcare services, including pharmaceuticals but also actively engaging communities to empower individuals in making informed health decisions. This engagement is critical, as it creates a sense of ownership over health outcomes and can lead to healthier behaviours at the community level. Equity ensures that healthcare is available and affordable for all, regardless of social status, with quality measures addressing disparities based on wealth, gender, or ethnicity (McCoy & Allotey, 2021). This value is particularly important in addressing disparities based on wealth, sex, or ethnicity, as equitable healthcare leads to improved overall health outcomes.

Resilience is essential for health systems to effectively respond to crises, such as pandemics or natural disasters, without compromising core functions. The COVID-19 pandemic has highlighted the necessity for health systems to be alert and responsive, revealing how systemic weaknesses can exacerbate health disparities. Efficiency emphasises the need to minimise waste and maximise health outcomes using available resources (Kruk et al., 2018). To guide the adoption of these values, frameworks like Donabedian’s model (structure-process-outcome) and the WHO’s ‘building blocks’ are frequently utilised. These frameworks highlight the importance of system foundations, care delivery processes, and measurable impacts, each essential for enhancing both quality and accountability (Bruce, 1990; Donabedian, 2005; Roberts et al., 2003; Taylor et al., 2014; Tunçalp et al., 2015; WHO, 2007). Systems that incorporate these elements tend to achieve better patient outcomes and greater responsiveness. An appropriate mix of facilities and providers, coupled with quality-centred service delivery models, is vital for maximising health outcomes. Effective care connections among these providers ensure a continuum of care that can significantly enhance patient experiences and outcomes (Costa-Font & Hernández-Quevedo, 2012).

## Impact of high-quality health systems

Top-tier health systems not only enhance outcomes and lower mortality and morbidity rates but also build trust in healthcare services (Knaul et al., 2018). Trust is a critical factor in healthcare; in Australia, individuals generally exhibit high confidence in healthcare providers (Mengistu et al., 2023), which encourages them to seek necessary care and adhere to medical advice. In contrast, Qatar, through a series of healthcare reforms, is actively working to build public trust in its rapidly developing healthcare sector, emphasising quality and accessibility (Goodman, 2015). This effort reflects an understanding that trust is integral to effective healthcare delivery and is essential for creating a culture of engagement between providers and patients.

Economically, high-quality healthcare systems enhance productivity by promoting population health and reducing unnecessary care, with strong financing models to protect against catastrophic expenses. The economic implications of healthcare quality in both Australia and Qatar cannot be overstated. Australia’s focus on efficient resource use has led to improved productivity and reduced healthcare costs through preventive measures (Australian Institute of Health and Welfare, 2018). In contrast, Qatar’s significant investment in health aims to minimise waste and enhance economic stability, ensuring that healthcare spending translates into better health outcomes.

User experience is another vital aspect that deserves emphasis. In Australia, the integration of services and an emphasis on patient-centred care contribute to positive experiences, with shorter wait times and respect for individual preferences (Sharp et al., 2016). In Qatar, ongoing reforms aim to enhance user experiences by improving access to services, including pharmaceuticals and ensuring that healthcare facilities meet international standards (Sharp et al., 2016). The ongoing shifts in both systems highlight a shared commitment to improving the patient journey, which is critical for creating a positive public perception of health services.

## Models of healthcare systems

Understanding the different models of healthcare systems within the context of health economics is important, particularly when it comes to identifying the perspectives of evaluations, including resource types, outcome measures, and their implications for patient care and cost-effectiveness. For policymakers, understanding whether a system aligns with Beveridge (e.g. UK-style public funding), Bismarck (insurance-based), or a hybrid model (e.g. Australia or Qatar) is vital for developing targeted interventions and assessing value for money (Ridic et al., 2012). Table 1 provides an overview of healthcare system models.

**Table 1.** Healthcare system models.

Model	Description	Examples
Beveridge (Study.com, 2024)	Government-owned facilities Services free at point of care Funded via taxation	United Kingdom, Spain
Bismarck (Study.com, 2024)	Mandatory social insurance Funded via payroll taxes Public/private provider mix	Germany, France
National Health Insurance (Study.com, 2024)	Single public payer Funded by taxes Providers may be private	Australia, Qatar, Canada, Taiwan
Out-of-pocket (Study.com, 2024)	Direct payment by patients Limited government /insurance role	Many low-income countries
Mixed models (Study.com, 2024)	Combination of above elements Often fragmented coverage	United States and other several countries

While these models influence the governance and funding frameworks of healthcare systems, the methods by which providers are compensated significantly affect care delivery, patient outcomes, and system sustainability.

Fee-for-service (FFS) remains one of the most widely used payment models worldwide, compensating providers based on the number of services they deliver. However, despite its popularity, FFS is often criticised for promoting inefficiencies and overutilisation, and resulting in fragmented care, particularly in high-income nations. Studies suggest that FFS may impede care coordination and overall cost-effectiveness (Wang et al., 2021). In contrast, alternative approaches like bundled payments and value-based healthcare (VBHC) seek to align financial incentives with patient-centred outcomes. Bundled payments enhance efficiency during care episodes, while VBHC incentivizes providers to improve health outcomes, especially in managing chronic illnesses and reducing hospital readmissions (Wise et al., 2022).

From a policy viewpoint, these new models signify more than just financial changes as they represent a shift toward outcome-oriented care and equitable access. Countries such as Australia are increasingly adopting value-based principles in public service delivery, particularly through outcome-driven funding for hospitals and Health Technology Assessment (HTA)-informed pharmaceutical policies. In Qatar, where FFS still predominates in the private sector, ongoing reforms provide an opportunity to integrate more outcome-focused strategies, particularly in the area of expanding national insurance and developing public-private partnerships. Table 2 presents an overview of the payment mechanisms used in healthcare systems.

**Table 2.** Payment mechanisms in healthcare systems.

Payment mechanism	Description	Implications
Fee-for-Service (Jia et al., 2021; European Observatory on Health Systems and Policies, 2024)	Payment per service High volume focus	May incentivize overuse Less coordinated care
Bundled Payments (Jia et al., 2021; European Observatory on Health Systems and Policies, 2024)	One payment for episode of care	Encourages integration and cost control
Value-Based Healthcare (Leao et al., 2023)	Payment linked to outcomes Patient-centred metrics	Promotes quality and prevention Requires data systems

In Australia, the National Health Insurance model strengthens a robust public healthcare system that prioritises accessibility and quality of care (Dixit & Sambasivan, 2018). This model facilitates a comprehensive approach to healthcare, where the integration of value-based initiatives focuses increasingly on patient outcomes and satisfaction. Experts argue that this emphasis on results create a culture of accountability and continuous improvement within the healthcare sector. Conversely, Qatar’s healthcare system uses a blend of public and private elements, influenced by its rapid development and investment in health infrastructure (Allianz, 2024; expatica, 2024). While this model has led to improved access and quality, it also presents challenges in ensuring that care remains equitable across different segments of the population. Without strong regulatory alignment or outcome-based incentives, there is a risk that this mixed model may continue to create access disparities and inconsistent care quality among different population groups.

### Approach

This commentary applies a comparative case analysis approach using the WHO health system building blocks framework (Mills et al., 2024; WHO, 2010), covering service delivery, the health workforce, health information systems, access to essential medicines, health financing, and leadership/governance. These areas were chosen because they are central to both health systems and correspond well with established approaches to comparative health policy analysis, especially for examining pharmaceutical governance, access, and equity.

The study draws on a selection of evidence sources based on their relevance, credibility, and policy importance. Although emphasis was placed on recent publications to capture ongoing reforms and current developments, foundational and seminal works were also included when they offered valuable historical or conceptual background. The sources include peer-reviewed research and grey literature, such as materials produced by the WHO, the Organization for Economic Co-operation and Development (OECD), the Australian Institute of Health and Welfare (AIHW), the Pharmaceutical Benefits Scheme (PBS), and the Qatar Ministry of Public Health (MOPH). Further information was supplemented through national health strategies, policy documents, health system reports, and materials focused on HTA.

The comparative analysis was organised around major themes directly related to pharmaceutical policy and health system performance, including governance, financing, access to medicines, value-based care, and HTA. This structure supports a systematic comparison of similarities, differences, challenges, and policy implications across the two health systems.

## Healthcare system overview

### Australia

Australia offers universal national public health coverage primarily funded through general taxation and the national Medicare programme, which guarantees access to essential health services for all citizens and permanent residents (Australian Government Department of Health and Aged Care, 2024c). About 55% of Australians also hold private health insurance (Australian Competition & Consumer Commission, 2023). While this dual system seems to enhance flexibility and alleviate pressure on public hospitals, it also raises important equity concerns in relation to whether private coverage may compromise the inclusive objectives of universal healthcare (Australian Government Department of Health and Aged Care, 2024c).

Governance is shared between federal and state governments: the Commonwealth funds Medicare and pharmaceuticals, while states and territory governments manage public hospitals. In 2021-22, the total health spending in Australia reached AU$241.3 billion, equating to around AU$9,365 per person (Australian Institute of Health and Welfare, 2023). During this period, government health spending rose significantly by approximately 10%, while non-government spending declined by nearly 2% (Australian Institute of Health and Welfare, 2023). This shift outlines a growing reliance on public funding to support healthcare access, particularly during challenging economic periods.

### Qatar

Qatar’s public healthcare system also offers universal coverage, primarily funded by the government, with services mainly provided through the Primary Health Care Corporation (PHCC) and Hamad Medical Corporation (HMC) (expatica, 2024). However, in contrast to Australia’s shared governance and varied payer mix, Qatar’s system is predominantly government-funded, relying significantly on hydrocarbon revenues. The annual health expenditure in Qatar is about US$6 billion (approximately 2.9% of GDP) (World Bank, 2022), which is relatively modest compared to other high-income countries. This highlights the need to build stronger health system resilience in the face of volatile oil prices and shifting population demographics.

While the system is generally well-resourced and not chronically congested due to a smaller population, certain areas, particularly tertiary care and specialised services, experience high demand and longer waiting times (expatica, 2024). The growth of private healthcare options, supported by employer-based insurance, has helped accommodate excess demand and improve access for insured expatriates (Allianz, 2024; DGA Group, 2025). This dual-track access model offers free public-sector services for citizens while expatriates rely on private insurance, which may inadvertently exacerbate inequities in medication adherence, particularly for chronic conditions.

Furthermore, although Qatar has made significant investments in facilities and infrastructure, its long-term care services and trained workforce capacity are still lacking. Insights from Australia, especially regarding aged care and rural health system design, could guide Qatar's evolving strategy. Policymakers should focus on expanding geriatric care training, increasing the number of long-term care beds, and implementing integrated care models. These gaps points to areas that require further health system development and assessment.

## Health system financing and insurance models

### Australia

Australia’s healthcare financing model is built on a mixed public-private structure, with Medicare serving as the national universal health insurance programme. Medicare ensures free or subsidised access to pharmaceuticals and hospital services, with funding primarily sourced from the federal government through the Medicare Levy (Australian Government Department of Health and Aged Care, 2024c).

Public hospitals, operated by state and territory governments, offer free treatment to patients. Additionally, individuals can opt for private health insurance. State and territory governments oversee public hospitals, while the Commonwealth provides support for pharmaceuticals and specialist services (Australian Institute of Health and Welfare, 2024; Australian Competition & Consumer Commission, 2023).

Around 55% of Australians have private health insurance, which is linked to quicker access to elective procedures and greater options for specialist care (Australian Competition & Consumer Commission, 2023). This dual model improves system flexibility and gives patients more choices, but it also creates equity challenges. People with private insurance often have quicker access to elective procedures and a wider range of providers. On the other hand, individuals with lower incomes or no private insurance might experience longer wait times or limited access to specialists. This situation highlights the challenge of ensuring equitable access when healthcare use still differs across income groups and regions.

The PBS provides subsidies for essential medications and regulates out-of-pocket costs, ensuring they remain affordable based on cost-effectiveness standards set by the PBAC (Australian Government Department of Health and Aged Care, 2024b). However, despite this comprehensive approach, inequities continue to exist, especially in access to specialists, rural service provision, and for individuals lacking private insurance. These issues indicate that universal coverage alone may not be enough if timely access and provider choice are still distributed unevenly. Future research and policy should examine how structural changes, such as outcome-based subsidies or incentives targeted to rural areas, could enhance both access and equity across the system.

### Qatar

Qatar’s health system is primarily government-funded, providing free services to Qatari citizens, including primary care, hospital treatment, and pharmaceuticals through HMC and PHCC (AlRuthia et al., 2025). In contrast to Australia’s public-private hybrid model, Qatar’s system is largely centralised and financed through oil and gas revenues. This reliance raises sustainability concerns as the country works to diversify its economy. Expatriates, who make up the majority of the population, experience fragmented healthcare access, relying on employer-sponsored insurance or out-of-pocket payments. This segmentation creates a dual burden: while citizens benefit from generous entitlements, expatriates may encounter inconsistent access to essential services and medications. This highlights the difficulty of delivering truly universal and equitable access when eligibility for healthcare benefits depends on nationality and employment status, rather than on population health needs.

In response to this challenge, Qatar has initiated reforms aimed at establishing a mandatory national health insurance scheme for all residents (International Trade Administration, 2022). The plan includes collaboration with private insurers, diversification of benefit packages, and improved access to both public and private services. By incorporating digital health technologies, such as telemedicine and electronic health records, Qatar seeks to enhance efficiency and transparency in care delivery (International Trade Administration, 2022). While these steps are promising, they will need careful regulation to prevent the growth of private insurance from worsening inequities and to ensure that essential healthcare entitlements remain available to all residents.

Qatar’s reforms reveal a tension familiar to Australia. While Qatar benefits from a strong centralised foundation, it should be cautious to avoid the pitfalls observed in other systems, where unregulated privatisation results in fragmentation and diminished continuity of care.

Although both countries have universal coverage frameworks, neither ensures equitable access in practice. In Australia, access varies based on income and insurance status, while in Qatar, it is influenced by nationality and employment. This indicates that mere coverage is not enough; access is shaped by the interplay of financing, regulation, and governance.

Australia’s lengthy experience with private insurance shows how variations in coverage arrangements can affect healthcare access and equity. The growth of private insurance in Qatar emphasises the necessity for transparent criteria and regulation that prioritises equity. Qatar, despite its centralised governance, should avoid repeating Australia’s mistakes by allowing private insurance to expand without clear minimum benefit standards and oversight for equity. At the same time, Qatar’s integrated digital infrastructure, e-health registries, and centralised reforms offer valuable insights for Australia, which continues to face fragmented services across its states and territories. Conversely, Australia’s strategies regarding telehealth, rural incentives, and HTA-integrated pharmaceutical pricing provide useful models for Qatar as it works on reforming its benefits and health financing.

Both systems illustrate that effective health financing relies not only on how funds are raised and who is covered, but also on how access is managed, regulated, and distributed fairly, an important takeaway for countries pursuing healthcare reform in varied sociopolitical contexts.

## Pharmaceutical policy and access to medicines

### Australia

Australia's pharmaceutical policy is directed by the National Medicines Policy, which seeks to provide prompt access to safe, effective, and reasonably priced medications while prompting quality medicine use and a sustainable medications sector (Care, 2022). In this context, the PBS enhances pharmaceutical access by ensuring that medications are affordable and widely available. Inclusion in the PBS necessities robust evaluation by the PBAC, which assesses both clinical efficacy and cost-effectiveness. Australia's PBS is often referenced worldwide as a prime example of a pharmaceutical reimbursement framework that incorporates HTA into pricing and coverage decisions (Harvey, 2005). This model not only promotes financial sustainability but also supports evidence-based prescribing, ensuring public funds are directed toward medications that deliver meaningful health outcomes. However, despite its theoretical equity, questions remain about the effectiveness of PBS subsidies in rural and underserved populations. Additionally, the rising costs of novel therapies, especially in oncology and rare diseases, threaten sustainability.

A major challenge is ensuring that Australia’s cost-effectiveness thresholds reflect wider social factors, including disease severity, rarity, unmet need, and the needs of marginalised groups. Ongoing debate focuses on whether these thresholds should be adjusted using equity-oriented modifiers, such as disease burden, unmet need, and potential impacts on vulnerable populations, which could make Australia’s HTA framework more inclusive (Wang et al., 2018). While Australia’s approach is often commended for its transparency, critics argue that strictly following HTA criteria can inadvertently disadvantage therapies for smaller patient populations, particularly people with rare diseases. This discussion points to the importance of balancing quantitative tools like QALYs with broader ethical and societal considerations when making pharmaceutical decisions.

### Qatar

MOPH centrally coordinates pharmaceutical policy, with HMC and PHCC responsible for its implementation in the public sector (AlRuthia et al., 2025; Fadhil et al., 2022). The national formulary directs procurement and prescribing, allowing Qatari citizens to access free or heavily subsidised medications, demonstrating strong governmental support for public health. However, the situation for expatriates, who make up the majority of the population, is quite different. Access for non-citizens is largely contingent on employer-sponsored insurance or out-of-pocket expenses at private pharmacies, leading to disparities in medication access and affordability. This dual-access system raises significant equity concerns. For example, expatriate workers with chronic conditions may face challenges in maintaining treatment adherence due to financial limitations, which could result in higher long-term health burdens and healthcare costs. As Qatar moves to reform its insurance system, ensuring fair pharmaceutical access for a population with diverse socioeconomic backgrounds while also protecting fiscal sustainability. Additionally, the lack of a mature HTA framework restricts Qatar’s capacity to evaluate value for money when approving or negotiating prices for expensive therapies (AlRuthia et al., 2025).

This situation highlights a more profound structural issue: in Qatar, access to medicines is influenced not just by clinical need but also by legal status and insurance class. Conversely, Australia’s PBS does not differentiate between citizens and non-citizens for essential medicines once they are enrolled in Medicare. However, both systems encounter equity trade-offs, Qatar’s stemming from population segmentation, and Australia’s arising from geographic and insurance disparities. This illustrates that having coverage does not ensure access unless affordability, continuity, and the ability to navigate the system are distributed equitably.

Qatar’s ongoing development of its HTA is distinct from Australia's more established system and may enable the integration of economic, clinical, and ethical factors throughout its development. Although Australia’s PBAC is considered a global benchmark, its focus on strict thresholds may restrict flexibility for innovative or rare therapies. In contrast, Qatar, free from legacy frameworks, can incorporate economic, clinical, and ethical criteria into its HTA methods from the outset, using Australia’s transparency while tailoring the approach to its own context.

However, Qatar encounters several challenges: the absence of a mature HTA process hampers price negotiations and formulary decisions, and there are currently no national limits on out-of-pocket drug costs in the private sector. In contrast, Australia imposes a cap of about AU$30 on patient co-payments through the PBS, offering more consistent protection against catastrophic health expenditures (Australian Government Department of Health and Aged Care, 2022). Additionally, Australia’s average HTA approval time is roughly 220 days, supported by clear pathways and published timelines (Australian Government Department of Health and Aged Care, 2024a). In comparison, Qatar lacks centralised timelines, resulting in inconsistent access speeds and a lack of transparency in the decision-making process.

Australia and Qatar implement distinctly different models for pharmaceutical access, yet both encounter challenges related to equity and sustainability. Australia’s PBS, grounded in a robust HTA framework, provides transparent and subsidised access to medications but faces issues with geographic disparities and limited adaptability for rare or high-cost treatments. In contrast, Qatar’s centralised system offers generous benefits for citizens but leaves expatriates to contend with fragmented and often expensive access. While Australia enforces caps on out-of-pocket costs and has established HTA timelines, Qatar currently lacks price caps in the private sector and has yet to formalise its HTA processes. However, this situation allows Qatar the opportunity to build an HTA system from scratch, potentially incorporating ethical and equity-sensitive criteria from the outset, rather than having to retrofit them later, as Australia has done. Both systems emphasise that having pharmaceutical coverage is inadequate without mechanisms to ensure equitable distribution, timely access, and affordability for all population groups.

## Value-based healthcare

### Australia

Australia's healthcare system is currently undergoing a transition from traditional FFS and bundled payment models toward VBHC, emphasising improved patient outcomes relative to resource utilisation (Ipsen & Sheppard 2024; Wise et al., 2022). This shift seeks to address three key issues, including efficient resource use, integrated care pathways, and reforming incentives to prioritise health outcomes over service volume, encouraging providers to focus on delivering quality care. This transition reflects a growing recognition that the quality of healthcare services is paramount, and that create better health outcomes can lead to more sustainable healthcare systems (Cattel & Eijkenaar, 2020; Woolcock, 2021).

A key component of this transition is the Australian Health Performance Framework (AHPF) (Australian Institute of Health and Welfare, n.d.), which serves as a vital compass for guiding the health of Australians. It allows policymakers and health professionals to navigate the complex landscape of health outcomes and system performance by providing a comprehensive tool for analysis. At its core, the AHPF analyzes health outcomes and determinants, helping to identify what influences health across diverse populations and regions in Australia. This analysis is grounded in critical indicators that measure variety of performance aspects, from mortality rates to access to healthcare services. These indicators can be assessed at national, state, territory, and local levels, offering a detailed understanding of how different areas and groups are faring in terms of health. This detailed analysis, however, uncovers systemic inequities in health outcomes, especially concerning Indigenous health and rural service delivery. It raises the question of whether measuring outcomes alone is adequate without implementing targeted equity interventions.

Beyond mere analysis, the AHPF comprises carefully selected indicators that serve as benchmarks for health performance. Each indicator is designed to capture specific aspects of health and healthcare, enabling a nuanced evaluation of progress and challenges within the system. Despite advancements in these frameworks, significant challenges remain. Australia’s mixed public-private system can create fragmentation in accountability, complicating the uniform implementation of VBHC principles. Additionally, data systems are inconsistent, particularly in the private sector, which limits the ability to track outcomes and conduct longitudinal evaluations. This fragmentation highlights a significant structural barrier to the uniform implementation of VBHC, particularly in mixed financing systems like Australia’s, where private sector participants may not have the incentives to align with national performance metrics.

Sustaining consistent VBHC implementation across payers and providers is difficult because incentives and reporting requirements differ. Moreover, if VBHC programmes do not include required equity indicators, they may be less able to identify and reduce disparities affecting underserved groups (Australian Government Department of Health and Aged Care, 2024c). As Australia expands its use of patient-reported outcome measures (PROMs) and patient-defined goals, it will be essential to ensure that what counts as ‘value’ incorporates the priorities and experiences of diverse patient populations, and that people with limited health literacy or restricted access to care are not penalised in outcome-based approaches. Overall, these issues underscore the challenge of matching patient-centred concepts of value with system-wide performance metrics.

### Qatar

Qatar's healthcare system is also transitioning toward VBHC, a shift that aligns with wider Middle East and North Africa trends (2021). Key drivers of VBHC adoption in Qatar include rising healthcare costs, economic pressures, and an aging population, necessitating a reassessment of existing healthcare models, which have been predominantly fragmented and focused on a FFS payment approach (2021). Despite recognising the potential advantages of VBHC, Qatar confronts numerous barriers, including reliance on traditional payment models, complex regulatory environments, difficulties in patient data management, and a lack of sufficient healthcare infrastructure and a skilled workforce (2021). As Qatar moves forward with this transformation, there is a significant opportunity to lower healthcare costs, enhance care quality, and promote healthier lifestyles, while accounting for region-specific factors that may impact the effective implementation of VBHC (2021).

Historically reliant on FFS models and centralised service delivery, Qatar acknowledges that merely expanding access is no longer adequate; the focus should now shift toward efficiency, quality, and population health impact. However, this transition occurs within a fragmented health system, where Qatari nationals experience different standards of care and access compared to expatriates. VBHC presents an opportunity to align incentives, standardise quality benchmarks, and address these disparities, but only if implemented with clear governance, transparent metrics, and robust digital infrastructure. Importantly, Qatar’s centralised health governance and integrated digital systems may provide a structural advantage over more federated models like Australia’s. This positions Qatar to develop outcome-based reforms from the ground up, thereby avoiding the legacy misalignments between funding and performance that are often seen in older systems.

Unlike Australia, where legacy structures create inertia, Qatar’s relative institutional flexibility may facilitate quicker integration of value-based reforms, particularly if they are connected to HTA-guided provider payment and formulary decisions. Nevertheless, significant challenges remain. Qatar’s emerging VBHC approach should reconcile public expectations for premium, government-funded services with the realities of private sector involvement and expatriate insurance models. This necessitates a sophisticated approach to VBHC in Qatar, one that acknowledges demographic diversity and incorporates risk adjustment for non-citizen populations. Such considerations are essential to ensure that incentives do not inadvertently exacerbate access gaps.

Qatar will need to create performance metrics that reflect the needs of both citizens and a diverse expatriate population. At the same time, outcome-based contracting will depend on more robust data systems, interoperability, and protections to ensure that value-based reforms do not narrow into cost-cutting, especially for patients who are costly to treat and have high care needs.

Australia’s journey in VBHC illustrates the challenges of reconciling fragmented public-private financing with outcome-oriented reforms. Despite having established frameworks like the AHPF, the lack of equity metrics and system-wide alignment of incentives restricts its potential for transformation. In contrast, Qatar’s centralised system provides it with a structural advantage; however, its success hinges on the proactive integration of equity, accountability, and inclusive outcome measures into its VBHC framework.

Australia can draw insights from Qatar’s cohesive digital health infrastructure, which enables centralised performance monitoring and may facilitate a more unified adoption of VBHC. On the other hand, Qatar could learn from Australia’s systematic approach to performance evaluation and patient-reported outcomes, especially if tailored to accommodate a multi-tiered population. Ultimately, both countries encounter the same fundamental issue: how to define ‘value’ in healthcare without sidelining vulnerable groups. Value-based reform must extend beyond mere efficiency to ensure that outcomes are equitable, data systems are interoperable, and incentives align with broader public health objectives.

## Health technology assessment (HTA)

HTA is a systematic approach that assess the clinical effectiveness, safety, and cost-effectiveness of health technologies and interventions (Trowman et al., 2023; van der Wilt & Oortwijn, 2022). It plays a significant role in health economics by informing policy decisions related to resource allocation and healthcare delivery. However, while the methodology can be standardised globally, its implementation needs to be adapted to fit local contexts, populations, and priorities.

### Australia

Australia has a well-established HTA system that incorporates clinical and economic evaluations into national decision-making processes. Important agencies, including the PBAC and the Medical Services Advisory Committee (MSAC), evaluate technologies based on criteria such as clinical efficacy, safety, cost-effectiveness, and, to a lesser degree, health equity (Australian Government Department of Health and Aged Care, 2024a). However, the extent to which HTA actively incorporates equity varies. While the PBAC considers factors like severity and unmet need, there is no mandated equity impact assessment. This limitation hinders its ability to address population-specific disparities, particularly in rural and Indigenous communities, where access remains suboptimal despite national entitlements.

Despite being regarded as a benchmark, the system has its shortcomings. For instance, while equity is recognised as essential, there is no formal obligation to assess cost-effectiveness across various population groups, highlighting the need to systematically assess how HTA-informed decisions affect different communities, including Indigenous populations.

Also, concerns have been raised regarding delays in decision-making, lack of transparency in pricing negotiations, and insufficient emphasis on patient-centred outcomes. These challenges suggest that institutional maturity might sometimes lead to rigidity, which could slow down responsiveness to new health needs or innovations (Australian Government Department of Health and Aged Care, 2024a). Nevertheless, Australia’s experience demonstrates that well-integrated HTA can enhance public trust and financial sustainability, particularly through its influence on the PBS. It also highlights the need for HTA systems to adapt and evolve, becoming more transparent, inclusive, and flexible to remain effective in a rapidly changing health environment. Australia’s experience highlights that HTA maturity alone does not guarantee adaptability or equity. Unaddressed institutional rigidity can impede responsiveness to novel therapies and marginalised populations. These are critical considerations for emerging systems like Qatar’s, which have the opportunity to embed flexibility and stakeholder inclusivity from the outset.

### Qatar

Qatar is in the early stages of establishing its HTA infrastructure. Led by the MOPH, recent initiatives aim to incorporate HTA into the procurement of pharmaceuticals and medical technologies, particularly as the country faces rising costs from advanced biologics and innovative therapies (Alnaqbi et al., 2024; Fasseeh et al., 2020). Initial efforts have involved collaborations with international HTA organisations and piloting methodologies adapted to Qatar’s specific context. Qatar’s early engagement with international HTA actors offers a unique opportunity to co-develop context-sensitive methods. Instead of retrofitting imported models, Qatar could focus on prioritising population-specific disease burdens and real-world affordability constraints in its value frameworks.

The development of Qatar’s HTA is timely, aligning with broader reforms aimed at value-based healthcare. However, challenges remain, including limited post-listing evaluations, gaps in data infrastructure, and the lack of formal price negotiation frameworks. Additionally, Qatar’s unique demographic structure, where citizens benefit from free public care while a large expatriate population relies on private insurance or out-of-pocket payments, complicates equitable HTA implementation. This dual population structure raises significant methodological and policy challenges for HTA. Defining value in a context where access and payer eligibility vary across population groups requires balancing both economic efficiency and equity. In addition, evaluating equity becomes more difficult when pricing, availability, and use differ based on residency status. This structural segmentation makes it essential for HTA in Qatar to be not only technically rigorous but also socially responsive, capable of reflecting both the economic priorities of the public system and the health needs of its uninsured expatriate majority.

Qatar's implementation challenges, including fragmented data streams, the absence of lifecycle pricing strategies, and ambiguous re-evaluation protocols, reflect the initial difficulties experienced by numerous HTA systems. Tackling these issues proactively can enable Qatar to bypass conventional implementation obstacles.

However, these challenges align with continuous efforts to develop HTA processes that are adaptable to the needs of both the public and private sectors. Variations in the health systems of Australia and Qatar show that HTA methods might need to be adapted to suit local circumstances. Qatar's developing HTA system features ongoing assessment of tiered evidence demands, advancements in digital health infrastructure, and strategies to support long-term viability. Qatar’s digital transformation initiatives, such as national e-health records, can facilitate real-world evidence generation, which is often a limitation in emerging HTA environments. By using centralised data, Qatar could design post-listing surveillance systems from the ground up, something that has proven challenging within Australia’s federated structure.

Assessing the economic implications of HTA practices in Australia and Qatar presents a valuable opportunity to explore how effective methodologies from Australia can be adapted and implemented in Qatar, whereby Australia's robust framework emphasises not only clinical and economic evaluations but also the importance of stakeholder engagement, ensuring that the needs of all involved parties are considered (Australian Government Department of Health and Aged Care, 2024a). This participatory approach could serve as an invaluable model for Qatar, facilitating the HTA process and acceptance of new health technologies among clinicians and the public.

For Qatar, the challenge lies not only in replicating Australia’s model but in adapting its principles to suit a unique demographic and health system context. With a population that includes a significant proportion of expatriates, who have diverse health needs and varying insurance coverage, HTA processes should be customised to reflect this complexity. Effective implementation will depend on Qatar's ability to develop flexible assessment pathways that accommodate different levels of evidence, usage patterns, and access constraints across various segments of the population.

Moreover, the epidemiological trends in Qatar differ from those in Australia, with unique health issues emerging from lifestyle factors and environmental conditions. Therefore, aligning the HTA process with local health priorities means identifying these trends and tailoring evaluations to focus on interventions that are most effective for the local context. For example, if a particular chronic disease is more prevalent in Qatar due to lifestyle factors, the HTA framework should prioritise technologies that target prevention and management of that condition.

Further, building capacity in HTA within Qatar is important for successful implementation. Investing in education and training for local healthcare professionals, policymakers, and economists will help facilitate a skilled workforce capable of conducting effective HTA. Collaborating with international experts, such as Australian experts in HTA (Australian Government Department of Health and Aged Care, 2024a), could also facilitate knowledge transfer and provide insights into best practices for effective implementation. Moreover, establishing an ongoing dialogue between stakeholders in both countries will be beneficial in identifying common challenges and sharing successful strategies. This cross-national partnership may enhance Qatar's HTA capabilities, resulting in enhanced health outcomes, more efficient use of resources, and greater public and stakeholder confidence in the health system. Australia’s centralised HTA model provides valuable insights into balancing rigour with stakeholder trust. Its experience demonstrates that merely embedding cost-effectiveness thresholds does not ensure access or public confidence. With its agile governance and growing digital infrastructure, Qatar has the opportunity to create a more inclusive system from the start, one that prioritises social equity, phased innovation adoption, and real-world impact. These factors highlight the necessity of tailoring HTA methods to align with the specific characteristics of local health systems and the needs of the population.

In the end, Qatar’s HTA journey reflects a wider global challenge: developing HTA approaches that work for health systems serving two distinct population groups, operating with limited local data infrastructure, and coping with growing expectations for evidence-based decisions. Solving this problem will require more than technical innovation, it will also depend on strong political commitment to reform that prioritises health equity and supports outcome-focused decision-making.

## Public health challenges

### Australia

Although existing models such as activity-based funding and global budgets have been introduced, the predominant FFS framework continues to drive high service volumes, often at the expense of value (Ipsen & Sheppard, 2024; Wise et al., 2022). This transition to VBHC faces several significant challenges, including financial risk distribution, structural barriers, data infrastructure, and provider engagement. Fairly distributing financial risk between providers and funders is crucial; without clear mechanisms for sharing this risk, there’s potential for conflict and inefficiency. Overcoming structural barriers inherent in Australia’s mixed public and private healthcare sectors is essential, as these barriers can hinder the integration of services and lead to fragmented care. Investing in robust data infrastructure is important for effectively tracking outcomes and costs, as accurate data collection and analysis are necessary for assessing the impact of VBHC initiatives. Engaging healthcare providers in the reform process is critical, ensuring that payment incentives are attractive enough to encourage participation and that providers are invested in delivering high-quality care (Cattel & Eijkenaar, 2020; Conrad, 2015; Woolcock, 2021). Addressing these complex financial, organisational, and data-related challenges is key to successfully implementing VBHC in Australia, ultimately shifting the focus toward delivering high-quality, patient-centred care.

Australia’s experience shows that while system maturity offers stability, it can also create structural inertia, slowing down reforms, especially in VBHC implementation across state-federal boundaries and in aligning private providers. These lessons are particularly pertinent for Qatar, where institutional flexibility may enable quicker adoption of reforms if coordinated effectively from the beginning.

It is important to acknowledge that care deemed ‘high quality’ by clinical or system standards may still not align with the individual needs, goals, or values of patients. Value-based healthcare seeks to bridge this gap by measuring outcomes that matter to patients, such as functional ability, quality of life, and emotional well-being, rather than focusing solely on procedural metrics or efficiency targets. However, this raises a critical policy dilemma: who defines patient-centred outcomes, and how are they prioritised across diverse population groups? Without the targeted inclusion of underserved communities in the collection and analysis of PROMs, VBHC could inadvertently reinforce existing inequities instead of addressing them. Qatar may benefit from embedding these patient voice mechanisms early in its transition. In this way, VBHC offers a more holistic approach that complements, but does not replace, traditional definitions of high-quality care.

### Qatar

Despite recognising the potential advantages of VBHC, Qatar confronts numerous barriers, including reliance on traditional payment models, complex regulatory environments, difficulties in patient data management, and a lack of sufficient healthcare infrastructure and a skilled workforce (2021, 2021). These barriers are further complicated by Qatar’s dual health system for citizens and expatriates, making it challenging to design unified outcome-based payment models. Equity concerns may emerge if VBHC implementation primarily focuses on public providers, potentially excluding private-sector expatriate services from quality and value reforms. While stakeholders recognise the importance of addressing NCDs, a comprehensive strategy for implementing VBHC is still lacking.

Here, existing literature provides several suggestions for future improvement within the public health systems of both Australia and Qatar. For instance, studies show that Australia could benefit from optimising preventive health strategies focused on reducing the incidence of lifestyle-related medical conditions, such as diabetes and obesity, through educational campaigns and community-based programmes (Australian Government Department of Health and Aged Care, 2021), and using broader outcome measures such as productivity adjusted-life years (PALYs) (Abushanab et al., 2024, 2025). The use of broader metrics like PALYs could guide Qatar in assessing the macroeconomic impact of NCDs. This is particularly relevant due to Qatar's younger workforce and its economic reliance on a healthy expatriate population. Integrating these metrics can help position prevention strategies as not just health priorities, but also as economic imperatives.

On the other hand, the literature highlights the need for Qatar to invest in the expansion of its long-term care services and facilities to better support an ageing population, while also implementing targeted public health interventions to curb diabetes rates (Planning and Statistics Authority, 2008). Moreover, both countries would benefit from integrated health policies that enhance collaboration between public and private healthcare sectors, leading to improved resource allocation and better overall health outcomes. Also, both countries, a critical future research and policy question arises: How can national VBHC reforms prioritise prevention, equity, and patient-centred design while addressing entrenched payment models and fragmented governance?

In both systems, the challenge is to align diverse stakeholders, payers, providers, and policymakers, around a shared definition of value. Australia’s struggles with reforming entrenched FFS incentives across jurisdictions highlight the importance of early alignment. For Qatar, its relatively centralised governance presents a unique opportunity to implement coordinated reforms, provided there is a commitment to transparency, stakeholder engagement, and data readiness.

### Limitations

While this commentary provides a structured comparison between two countries, it has several limitations. First, focusing on just two countries restricts the generalizability of the findings to other healthcare systems. Second, the analysis relies on secondary literature and public policy documents, lacking insights from stakeholder interviews or direct implementation data. Third, the absence of real-world metrics or case studies limits the assessment of how proposed reforms are put into practice. Despite these limitations, the comparative insights can guide future empirical research and policymaking in similar contexts.

## Future directions and policy implications

### Australia

Policymakers should address significant disparities in access and outcomes, particularly for rural and Indigenous populations (Baazeem et al., 2024). Rural areas often face a scarcity of healthcare providers and facilities, leading to inequitable access to essential services, including pharmaceuticals. Indigenous Australians experience markedly poorer health outcomes compared to non-Indigenous populations, necessitating targeted initiatives to close these gaps (Baazeem et al., 2024). At the same time, Australia’s escalating mental health crisis, worsened by socioeconomic disparities, highlights the pressing need for a transition to preventive care, increased adoption of digital health solutions, and enhanced collaboration among healthcare, education, and social services (Hashmi et al., 2023; van Gaans & Dent, 2018). Integrated data systems and telehealth platforms could help close service gaps, especially in remote areas. However, implementing these innovations should be paired with safeguards to ensure they do not worsen existing inequities.

While cost containment will remain a priority, policymakers need to proceed with caution: strict budget cuts without a strategic plan risk negatively impacting health outcomes. Preventive care, when backed by coherent funding and delivery systems, can reduce long-term costs while improving quality of life. Thus, Australia’s policy focus should evolve from reactive, volume-driven care to proactive, value-oriented investment. A critical element of this transition may be co-design, engaging not just clinicians and economists but also patients and communities in defining what ‘value’ means.

### Qatar

To facilitate the transition to VBHC, it is important to increase awareness of VBHC among all stakeholders, establish a coordinated policy framework, test innovative payment approaches, and develop robust data systems for measuring costs and outcomes. As Qatar moves forward with this transformation, there is a significant opportunity to lower healthcare costs, enhance care quality, and promote healthier lifestyles, while accounting for region-specific factors that may impact the effective implementation of VBHC (2021, 2021).

Ultimately, both nations encounter similar pressures, rising costs, chronic disease burdens, and high public demands for quality and transparency. However, their circumstances require different approaches. In relation to Australia, the challenge lies in recalibrating and refining equity measures. For Qatar, the focus is on building a coherent system and institutional framework. Future comparative research could explore how policy experimentation in emerging systems like Qatar can inform reforms in more established systems like Australia, and vice versa.

## Policy transferability and global relevance

The comparison between Australia and Qatar reveals potential avenues for selective policy transfer, particularly in pharmaceutical governance and HTA. Australia’s HTA system, backed by well-established institutions like the PBAC and MSAC, exemplifies centralised, evidence-driven decision-making. However, direct application in Qatar may face limitations due to specific contextual factors. Qatar’s distinct demographic landscape, dominated by expatriates, fragmented insurance coverage, and a nascent HTA framework, requires bespoke approaches. Nevertheless, concepts such as stakeholder engagement, transparent pricing, and tiered evidence requirements could be adapted to align with Qatar’s health system objectives. Furthermore, Australia’s challenges, including rigid cost-effectiveness thresholds and overlooked equity concerns, offer valuable lessons for Qatar as it develops its own HTA processes.

Several enabling factors could enhance this transferability. Qatar’s centralised governance and national digital health strategy may facilitate quicker implementation of HTA reforms compared to Australia’s more decentralised health system. Collaborative efforts, including cross-national technical exchanges, investments in local expertise, and structured pilot programmes, could strengthen Qatar’s ability to adapt and implement components of Australia’s regulatory and assessment frameworks.

Beyond the Australia-Qatar comparison, this analysis provides insights into broader global health system challenges. Rising medication costs, the pursuit of sustainable universal health coverage (UHC), and the need to balance innovation with equity are pressing issues affecting both high-income and transitioning health systems. The WHO’s strategies regarding access to essential medicines, equitable pricing, and enhancing HTA capacity align with the themes explored in this commentary. As countries navigate post-pandemic recovery, inflationary pressures, and demographic shifts, frameworks that integrate cost-effectiveness, stakeholder engagement, and equity considerations will become increasingly essential. The lessons from Australia and Qatar contribute to a wider discourse on how nations can develop more sustainable, inclusive, and accountable pharmaceutical and HTA policies (Table 3).

**Table 3.** A comparative summary using World Health Organization building blocks.

Domain	Australia	Qatar
Governance	Federal and state-level health responsibilities HTA and pricing committees (PBAC, MSAC) Strong regulatory frameworks 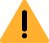	Centralised through MOPH Emerging HTA committee 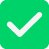 Public-sector governance
Health Financing	Mixed system (Medicare + private) Public funding via taxation Safety nets for OOP costs 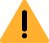	Public fundingfor citizens Insurance-based and OOP for expatriates 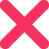 Insurance reform underway 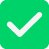
Service Delivery	Universal access (Medicare) Geographic disparities in rural/remote areas 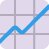 Integrated public-private care pathways	Free/subsidised care for citizens Expatriates reliant on employer/private systems 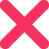 Limited continuity across providers
Health Workforce	Highly trained workforce Rural access challenges 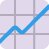 Strong academic pipeline	Diverse expatriate workforce Capacity gaps in public health, HTA 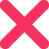 Training investments underway 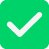
Health Information Systems	Robust public-sector digital infrastructure Data silos in private settings 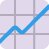 PROMs/PREMs increasingly used	National EHR (Cerner) in HMC/PHCC Fragmentation between public/private 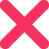 PROMs integration nascent 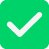
Access to Essential Medicines	PBS ensures affordability HTA-guided inclusion 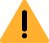 Limited flexibility for ultra-rare/oncology drugs 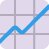	Central procurement for public hospitals Citizen coverage; limited expatriates coverage 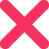 HTA-guided selection in progress 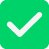
HTA and Innovation	Mature HTA process via PBAC/MSAC Cost-effectiveness threshold used Equity inclusion limited 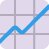	HTA emerging via MOPH Limited use in pricing/negotiation 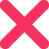 Pilots with WHO & partners 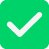

HTA: Health Technology Assessment; PBAC: Pharmaceutical Benefits Advisory Committee; MOPH: Ministry of Public Health; OOP: Out-of-Pocket; VBHC: Value-Based Healthcare; MSAC: Medical Services Advisory Committee; PROMs: Patient-Reported Outcome Measures; PREMs: Patient-Reported Experience Measures; EHR: Electronic Health Record; PHCC: Primary Health Care Corporation; HMC: Hamad Medical Corporation; PBS: Pharmaceutical Benefits Scheme; WHO: World Health Organization.

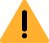
 =  Established / functional; 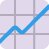 =  Partial / in transition;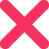 =  Not in place / absent; 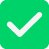 =  Emerging / in development.

## Conclusion

This commentary compared Australia and Qatar to examine how their differing health system structures impact pharmaceutical policy, the adoption of HTA, and access to equitable care. Australia features a well-established, institutionalised model centred on HTA and value-based care, while Qatar offers a more adaptable system that allows for the development of customised reforms. Three key policy insights emerge from this comparison: (i) (i) HTA can support clear and evidence-based decision-making when tailored to specific local settings; (ii) equity factors are important for addressing the needs of underserved groups, such as Indigenous and rural individuals in Australia and expatriate communities in Qatar; and (iii) a strong health data framework is important for enabling value-based healthcare and assessing policy effectiveness. The experiences of the two nations highlight the significance of context-aware policy formulation and the benefits of learning from each other in addressing common issues related to healthcare accessibility, cost-effectiveness, and sustainability. Future research should further explore stakeholder perspectives and system readiness, especially in emerging HTA settings.
